# Plasticity of GABA transporters: an unconventional route to shape inhibitory synaptic transmission

**DOI:** 10.3389/fncel.2014.00128

**Published:** 2014-05-13

**Authors:** Annalisa Scimemi

**Affiliations:** Department of Biology, SUNY AlbanyAlbany, NY, USA

**Keywords:** GABA, GABA transporters, GAT1, GAT3, uptake, diffusion, spillover, synaptic transmission

## Abstract

The brain relies on GABAergic neurons to control the ongoing activity of neuronal networks. GABAergic neurons control the firing pattern of excitatory cells, the temporal structure of membrane potential oscillations and the time window for integration of synaptic inputs. These actions require a fine control of the timing of GABA receptor activation which, in turn, depends on the precise timing of GABA release from pre-synaptic terminals and GABA clearance from the extracellular space. Extracellular GABA is not subject to enzymatic breakdown, and its clearance relies entirely on diffusion and uptake by specific transporters. In contrast to glutamate transporters, GABA transporters are abundantly expressed in neuronal pre-synaptic terminals. GABA transporters move laterally within the plasma membrane and are continuously trafficked to/from intracellular compartments. It is hypothesized that due to their proximity to GABA release sites, changes in the concentration and lateral mobility of GABA transporters may have a significant effect on the time course of the GABA concentration profile in and out of the synaptic cleft. To date, this hypothesis remains to be tested. Here we use 3D Monte Carlo reaction-diffusion simulations to analyze how changes in the density of expression and lateral mobility of GABA transporters in the cell membrane affect the extracellular GABA concentration profile and the activation of GABA receptors. Our results indicate that these manipulations mainly alter the GABA concentration profile away from the synaptic cleft. These findings provide novel insights into how the ability of GABA transporters to undergo plastic changes may alter the strength of GABAergic signals and the activity of neuronal networks in the brain.

## Introduction

There is a population of neurons in the mammalian brain that differs for their morphology, embryonic origin, connectivity and firing properties, but that shares the common ability to synthesize GABA, transport it into synaptic vesicles and release it in the synaptic cleft to communicate with post-synaptic target cells (Defelipe, 1993; Cauli et al., 1997; Gupta et al., 2000; Ascoli et al., 2008; Klausberger and Somogyi, 2008; Vitalis and Rossier, 2011). GABAergic neurons control the onset of large-scale network oscillations at various frequency ranges during development and in the mature brain, and their dysfunction is implicated with the onset of disease states like epilepsy, schizophrenia and autism (Le Magueresse and Monyer, 2013). In order to coordinate the activity of large neuronal ensembles, it is necessary to perfectly time GABA release from pre-synaptic terminals with GABA receptors activation in pre- and post-synaptic membranes, and GABA clearance from the extracellular space. There is no enzyme in the extracellular space that can convert GABA into a biologically inert molecule. As a consequence, GABA clearance relies entirely on diffusion and uptake by specific GABA transporters. As GABA diffuses away from its release site, it binds to synaptic and extra-synaptic receptors and to GABA transporters. Despite their name, GABA transporters do not always translocate across the cell membrane all the GABA molecules that they bind (i.e., they do not have 100% transport efficiency) but, in some cases, they can also release them back in the extracellular space (Bicho and Grewer, 2005). These events are reminiscent of those experienced by other neurotransmitters that are not subject to extracellular enzymatic degradation, like glutamate (Bergles et al., 1999).

One key difference, however, is that GABA and glutamate transporters have different cellular and sub-cellular distributions and different levels of expression (Zhou and Danbolt, 2013). With the exception of thalamic, Purkinje and striatonigral synapses, the highest level of expression of GABA transporters is found in neurons (Zhou and Danbolt, 2013). In contrast, glutamate transporters are abundantly expressed in astrocytes (Danbolt, 2001). In the hippocampus, the density of expression of GABA transporters is 800–1300 μm^−2^ (Chiu et al., 2002), considerably lower than that of glutamate transporters (10,800 μm^−2^ Lehre and Danbolt, 1998). This scarceness of GABA transporters could increase the likelihood of GABA spillover over that of glutamate in this brain region.

There are two main types of GABA transporters in the brain: GAT1 and GAT3. Neurons express GAT1, whereas astrocytes express GAT1 and GAT3 (Minelli et al., 1995, 1996). Immunocytochemistry experiments indicate that in neurons, GABA transporters are mainly localized in pre-synaptic GABAergic axon terminals (Radian et al., 1990; Ikegaki et al., 1994; Minelli et al., 1995; Conti et al., 1998; Zhou and Danbolt, 2013). One may consider this to be a strategic location, because it is the closest to GABA release sites. At excitatory synapses, glutamate transporters are located further away from the release sites, mainly in astrocytic processes adjacent to active synapses (Danbolt, 2001; Scimemi et al., 2009; Holmseth et al., 2012; Zhou and Danbolt, 2013). It is unclear whether the location of GABA transporters allows them to clear the released neurotransmitter more effectively than glutamate transporters. This could happen if the GABA transporters present inside the synaptic cleft were many and with rapid binding kinetics (see also Rusakov et al., 2011). It remains unclear whether GABA transporters can shape the GABA concentration profile inside the synaptic cleft, given what is currently known about their expression, binding kinetics, and transport efficiency.

Several experimental findings converge to indicate that GABA transporters in the cell membrane constitutively recycle to/from the cytoplasm (Deken et al., 2003; Wang and Quick, 2005) and move laterally within the lipid bilayer (Imoukhuede et al., 2009; Moss et al., 2009). Both phenomena are considered to be “rapid.” The time constants of GABA transporters exo/endocytosis are 1.6 and 0.9 min, respectively (Wang and Quick, 2005) and the fluorescence recovery after photobleaching of surface expressed GAT1-YFP8 molecules occurs with a half time of ~20 s (Imoukhuede et al., 2009). There are intracellular signaling cascades that can alter the number of GABA transporters expressed in the cell membrane and their recycling rate toward the cytosol (Whitworth and Quick, 2001a,b; Deken et al., 2003; Wang and Quick, 2005). Accordingly: PKC activation and tyrosine kinase inhibition cause a reduction in GABA uptake (Beckman et al., 1998, 1999; Law et al., 2000); depolarizing events that induce activation of voltage-gated Ca^2+^ channels increases the recycling rate of GABA transporters (Deken et al., 2003); proteins of the SNARE complex that mediate neurotransmitter vesicle release, like syntaxin 1A, interact with GABA transporters and increase their surface expression (Beckman et al., 1998; Deken et al., 2000). These findings provide evidence that the neuronal expression of GABA transporters can be modified within and across synapses depending on their level of activity. Likewise, the mobility of GABA transporters within the cell membrane can also be regulated by intracellular signaling cascades that involve PKC activation and that alter the interaction between GABA transporters and adapter proteins that anchor them to the cell cytoskeleton (Imoukhuede et al., 2009; Moss et al., 2009).

What is the effect of these modifications? How does the GABA concentration profile in the synaptic cleft and in the surrounding extracellular volume change, with different levels of expression and mobility of GABA transporters? Here we address this question by using 3D Monte Carlo reaction-diffusion simulations of GABA release from an active synapse. Our findings indicate that: (1) varying the concentration of GABA transporters alters activation of GABA receptors away from the release site, not of GABA receptors in the post-synaptic membrane directly opposed to it; (2) increasing the lateral mobility of GABA transporters facilitates GABA diffusion away from the synaptic cleft without altering the lifetime of GABA in the extracellular space. We analyze these effects during single and repeated stimulations. Taken together, these findings indicate that by altering the expression and diffusion of GABA transporters, the brain can control, in an activity-dependent manner, the spatial specificity of GABAergic signals.

## Model description: geometry and settings

**Figure 1:**
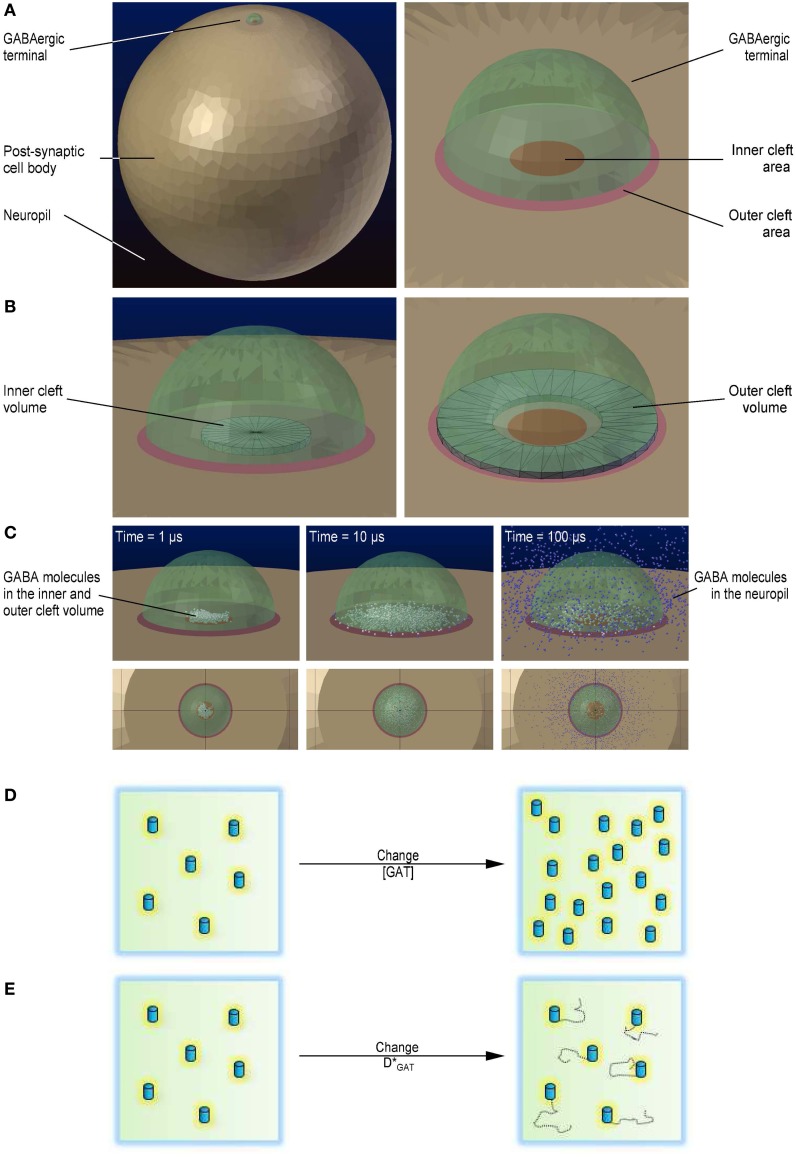
**3D geometry of the modeled simulation environment. (A)** Geometry of the simulation environment used to run the Monte Carlo reaction-diffusion simulations in CellBlender (left). The large light brown sphere represents the cell body of a post-synaptic cell. The small green, semi-transparent hemisphere represents an axo-somatic GABAergic synaptic terminal. The dark blue background represents the neuropil. Close-up view of the presynaptic terminal, including the inner and the outer cleft areas (right). The red circle represents the inner cleft area. The pale red annulus represents the outer cleft area. **(B)** The black wireframe shows the portion of the synaptic cleft volume above the inner cleft area in which we monitored the GABA concentration (left). The black wireframe shows the portion of the synaptic cleft volume above the perisynaptic region in which we monitored the GABA concentration (right). **(C)** Localization of GABA molecules diffusing away from their release site. GABA molecules diffusing within the inner and outer cleft volume are represented as white spheres. GABA molecules diffusing in the neuropil are represented as blue spheres. The three snapshots were obtained 1 μs (left), 10 μs (middle) and 100 μs after release (right). **(D)** Schematic diagram illustrating the change in GABA transporter concentration. **(E)** Schematic diagram illustrating the change in GABA transporter surface mobility.

**Figure 2:**
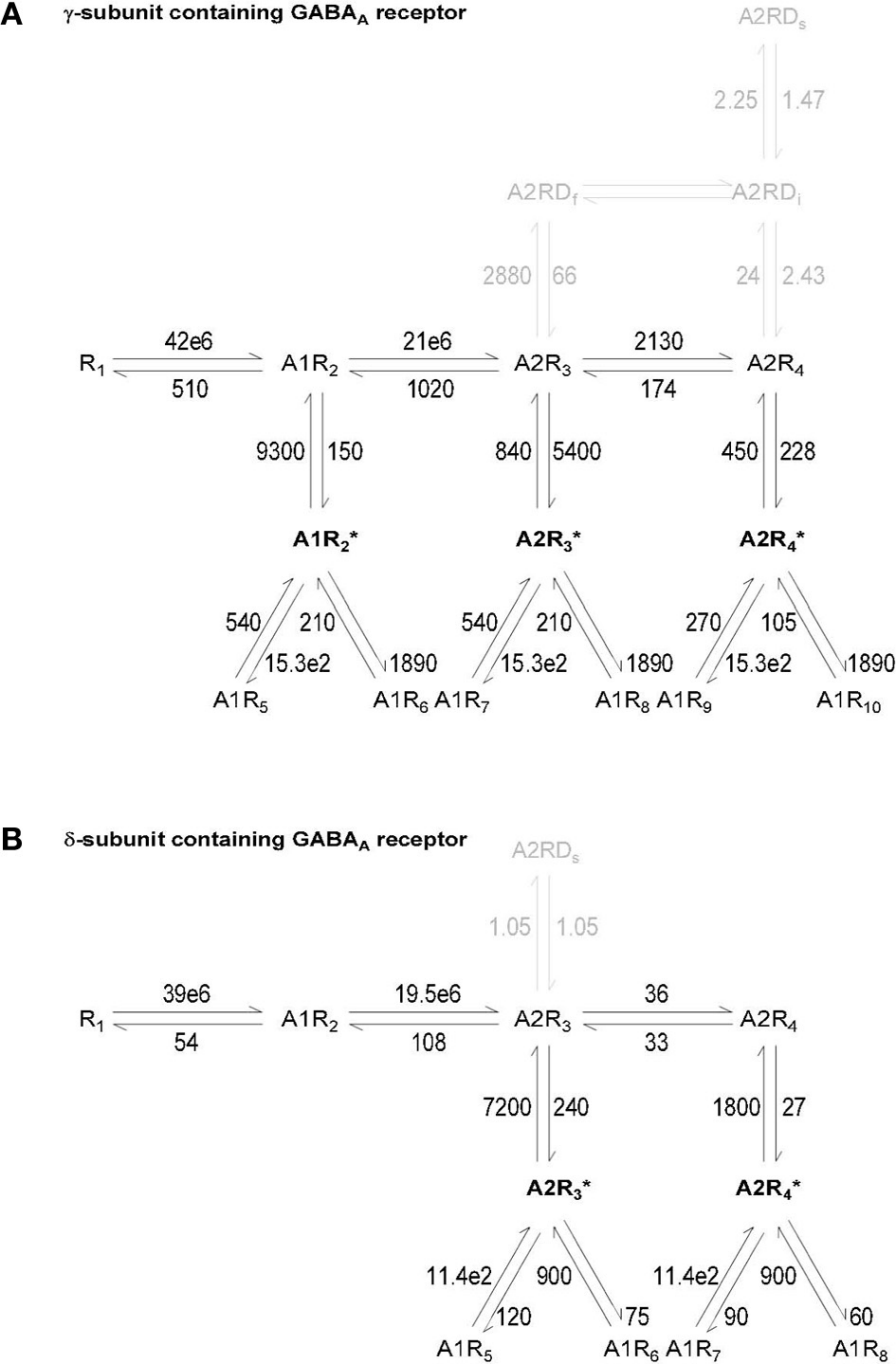
**Kinetic model of γ- and δ-subunit containing GABA_A_ receptors**. The kinetic models of *γ*-subunit containing GABA_A_ receptors **(A)** and δ-subunit containing GABA_A_ receptors **(B)** correspond to the ones developed by Haas and Macdonald (1999) and were corrected for a *Q*_10_ = 3 (Gonzales et al., 2007). Agonist molecules are indicated by A, resting states of the receptor by R, desensitized states by D and open states by an asterisk. *D*_*f*_, D_*i*_ and D_*s*_ represent the fast, intermediate and slow desensitized states of γ-subunit containing GABA_A_ receptors. The *δ*-subunit containing GABA_A_ receptors only have a *D*_*s*_ state. The units for all rate constant are s^−1^ except for GABA binding states, which are expressed in M^−1^s^−1^.

We used Blender 2.69 to design a simulation environment containing the 3D geometry of an average mouse hippocampal *stratum radiatum* axo-somatic GABAergic synapse (estimated by comparing the synaptic structure analysis from Nusser et al., 1997; Schikorski and Stevens, 1997; Biro et al., 2006; Specht et al., 2013). Figure 1 provides an overview of the geometry of the simulation environment created *in silico* with Blender (Figures 1A–C), together with a schematic representation of the parameters that were tested (Figures 1D–E). The simulation environment consisted of a cube (11 μm wide), which we refer to as the “world.” The world had a volume of *V*_*world*_ = 1331 μm^3^ and contained the soma of an ideal post-synaptic cell and the pre-synaptic terminal of an ideal GABAergic bouton. The portion of the world that was not occupied by the soma and the pre-synaptic terminal was referred to as the neuropil. The soma was represented as a sphere with the radius (*r*) of a typical hippocampal *stratum radiatum* interneuron (*r*_*post*_ = 5 μm). The pre-synaptic terminal was represented as a hemisphere (*r*_*pre*_ = 0.3 μm). The inner cleft area was modeled as a circle (*r*_*icleft*_ = 0.1 μm) at the surface of the soma. The size of the inner cleft area matched the average size of the active zone region at small excitatory and inhibitory central synapses (Nusser et al., 1997; Schikorski and Stevens, 1997; Biro et al., 2006; Specht et al., 2013). The outer cleft area, which corresponds to the perisynaptic portion of the post-synaptic membrane, was represented as an annular region that extended for *r*_*ocleft*_ = 0.2 μm beyond the edge of the inner cleft area. We used CellBlender v1.0 (www.mcell.org) to simulate GABA release from the pre-synaptic terminal and diffusion in the extracellular space. At the beginning of each simulation, *n*_*GABA*_ = 2000 GABA molecules were released from the center of the flat region of the pre-synaptic terminal, in the inner volume of the synaptic cleft. When we monitored the effect of varying the density of expression of GABA transporters, we repeated each simulation for *n*_*seed*_ = 100 times; each simulation consisted of *n*_*iter*_ = 50,000 iterations with a time step of Δ*t* = 1 μs (i.e., a total simulation time of 50 ms). Each simulation required a significantly longer computational time when we monitored the effect of varying the diffusion coefficient of GABA transporters. These simulations were repeated for *n*_*seed*_ = 30 times and each simulation consisted of *n*_*iter*_ = 5000 iterations with a time step of Δ*t* = 10 μs (i.e., a total simulation time of 5 ms). We measured the free GABA concentration in the inner and outer cleft volume and in the neuropil. The GABA waveforms obtained in CellBlender were exported into ChanneLab2 (www.synaptosoft.com) to simulate the response of GABA_A_ receptors. The majority of native GABA_A_ receptors are thought to assemble as combinations of αβγ (here termed γ-subunit containing GABA_A_ receptors) or αβδ subunits (here termed δ-subunit containing GABA_A_ receptors) (Haas and Macdonald, 1999). There is evidence that δ-subunit containing GABA_A_ receptors are mainly extra-synaptic, whereas *γ*-subunit containing GABA_A_ receptors are present in synaptic and extra-synaptic regions (Nusser et al., 1998). Therefore, in our analysis, we compared the response of γ-subunit containing GABA_A_ receptors in the inner cleft area and of γ- and δ-subunit containing GABA_A_ receptors in the outer cleft area (Kasugai et al., 2010). The kinetic models for GABA binding to these receptors was taken from (Haas and Macdonald, 1999) and were corrected for temperature dependence using a *Q*_10_ = 3 (Gonzales et al., 2007), to obtain a more faithful representation of GABA_A_ receptor activation at physiological temperature. The kinetic models of γ- and δ-subunit containing GABA_A_ receptors are shown in Figure 2. A beta version of CellBlender was used to simulate repeated release events (Figure 4). A summary of all the simulation parameters in reported in Table 1.

**Table 1:** **Parameters used for the 3D Monte Carlo reaction-diffusion simulations**.

**Parameter**	**Abbreviation**	**Value**	**References**
World volume	*V*_*world*_	1331 μm^3^	
Radius of the post-synaptic soma	*r*_*post*_	5 μm	Ascoli et al., 2008
Radius of the pre-synaptic terminal	*r*_*pre*_	0.3 μm	Nusser et al., 1997; Specht et al., 2013; cf. Schikorski and Stevens, 1997
Radius of the inner cleft (i.e., radius of the active zone and of the inhibitory post-synaptic density)	*r*_*icleft*_	0.1 μm	Biro et al., 2006; Specht et al., 2013; cf. Kasugai et al., 2010
Radius of the outer cleft (i.e., radius of the peri-synaptic annulus)	*r*_*ocleft*_	0.2 μm	Nusser et al., 1997; cf. Schikorski and Stevens, 1997
Number of GABA molecules released	*n*_*GABA*_	2000	
Extracellular volume fraction	α	0.15	Scimemi et al., 2009
Tortuosity	λ	1.45	Scimemi et al., 2009
Free GABA diffusion coefficient (cf. glutamine)	*D*_*free*_	0.76 μm^2^/ms	Longsworth, 1953
Apparent GABA diffusion coefficient in the cleft	D^*^_cleft_	0.51 μm^2^/ms	
Apparent GABA diffusion coefficient in the neuropil	D^*^_world_	0.36 μm^2^/ms	
GAT binding rate	*k*_*on*_	5.9·10^6^ M^−1^s^−1^	Bicho and Grewer, 2005
GAT unbinding rate	*k*_*off*_	58.4 s^−1^	
GAT steady-state affinity	*k*_*m*_	12.1·10^−6^ M	Bicho and Grewer, 2005
GAT turnover rate	*k*_*cycle*_	13 s^−1^	Bicho and Grewer, 2005
GAT temperature dependence	*Q*_10_	3	Gonzales et al., 2007
GAT1 density in the pre-synaptic terminal	[*GAT1*]_*pre*_	650 μm^−2^	Chiu et al., 2002
GAT1 density in the neuropil	[*GAT1*]_*neuropil*_	3720 μm^−1^	Chiu et al., 2002
GAT3 density in the neuropil	[*GAT3*]_*neuropil*_	372 μm^−3^	
Simulations time step	Δ*t*	1–10 μs	
Simulations iterations	*n*_*iter*_	5000–50,000	
Simulations seeds	*n*_*seed*_	30–100	

## Model description: diffusion properties

We previously used an electron microscopy analysis to estimate the extracellular volume fraction of the mouse *stratum radiatum* hippocampal neuropil (α = 0.15) and integrative optical imaging and two-photon laser scanning microscopy analysis to estimate the tortuosity value in this region of the brain (λ = 1.45) (Scimemi et al., 2009). The measure of λ that we obtained with this approach includes a geometric (λ_*g*_) and a viscous component (λ_*v*_), where λ = λ_*g*_ · λ_*v*_. The geometric component describes the hindrance to diffusion by cellular processes and by cell membrane invaginations that create dead-end routes (Hrabetova et al., 2003; Hrabetova and Nicholson, 2004; Kinney et al., 2013); the viscous component describes the hindrance to diffusion due to the presence of charged, long-chain molecules in the extracellular matrix that drag neurotransmitters as they travel in the neuropil. The relationship between λ_*g*_ and α, is:

$$\lambda_{g}=\sqrt{\frac{3-\alpha }{2}}$$

(see also Tao and Nicholson, 2004). In this equation, α = 0.15 (see above) and therefore λ_*g*_ = 1.19. From the expression λ = λ_*g*_ · λ_*v*_ we estimated λ_*v*_ = 1.22. We reasoned that the viscous component of the tortuosity is the main factor that hinders neurotransmitter diffusion inside the synaptic cleft, where there are no cell process that create physical obstacles to diffusion (Barbour, 2001). The apparent diffusion coefficient (D^*^) is defined as D^*^ = *D*_*free*_/λ^2^. We approximated the value of the apparent diffusion coefficient in the cleft (D^*^_cleft_) to D^*^_cleft_ = *D*_*free*_/λ_*v*_, the free diffusion coefficient for GABA (*D*_*free*_) with the free diffusion coefficient for glutamine (*D*_*free*_ = 0.76 μm^2^/ms) (Longsworth, 1953), and estimated D^*^_cleft_ = 0.51 μm^2^/ms. The diffusion coefficient in the neuropil (D^*^_neuropil_) was estimated as D^*^_neuropil_ = *D*_*free*_/λ (i.e., 0.36 μm^2^/ms), and was in close agreement with the diffusion coefficient for glutamate derived experimentally by Nielsen et al. (2004).

## Model description: GABA transporter kinetics and density of expression

The kinetics of GABA transporters (GATs) was modeled according to the following reaction scheme:

(1) $$\text{GAT}+{\text{GABA}}_{\text{out}}\overset{k_{on}}{\underset{k_{off}}{↔}}\text{ GAT}-\text{GABA }\overset{k_{cycle}}{\to }\text{GAT}+{\text{GABA}}_{\text{in}}$$

The scheme includes a rapid and reversible GABA binding step and a slow and unidirectional translocation step, analogous to the one used to simulate the activity of glutamate transporters at excitatory synapses (Barbour, 2001; Diamond, 2001, 2005; Scimemi et al., 2009). In this simplified scheme, GABA transporters do not operate in the reverse mode [i.e., they do not release GABA from the cytosol to the extracellular space (Heja et al., 2012; Kirischuk et al., 2012)]. The rate of GABA binding to GAT1 (*k*_*on*_) was set to 5.9·10^6^ M^−1^ s^−1^ (Bicho and Grewer, 2005) and the unbinding rate (*k*_*off*_) was derived using the law of conservation of mass, whereby *k*_*off*_ = *k*_*on*_ · *k*_*m*_ − −*k*_*cycle*_ = 58.4 s^−1^. In this equation, *k*_*m*_ and *k*_*cycle*_ represent the steady-state apparent affinity for GABA (*k*_*m*_ = 12.1·10^−6^ M) and the turnover rate of GAT1 (*k*_*cycle*_ = 13 s^−1^), respectively (Bicho and Grewer, 2005). All rates were multiplied by *Q*_10_ = 3 to account for the temperature dependence of the reactions and describe their behavior at physiological temperature (Gonzales et al., 2007). Previous work on knock-in mice expressing GFP-tagged GAT1 has shown that the density of GAT1 expression in pre-synaptic boutons of GABAergic hippocampal interneurons is 800–1300 μm^−2^ (Chiu et al., 2002). According to this study, only 61–63% of these molecules are expressed on the plasma membrane, leading to an estimated density of expression of GAT1 on the cell membrane of pre-synaptic boutons of 496–806 μm^−2^. In our simulations, we set the density of expression of GAT1 on the cell membrane of the pre-synaptic bouton in control conditions to [*GAT1*]_*pre*_ = 650 μm^−2^, which corresponds to the mid-range of the available experimental estimates. The study by Chiu et al. (2002) also indicates that the density of expression of GAT1 in the whole hippocampal neuropil is 6000 μm^−3^. In our simulations, the control density of expression of GAT1 in the neuropil was set to [*GAT1*]_*neuropil*_ = 3720 μm^−3^ (i.e., 62% of 6000 μm^−3^). Because GAT3 is only expressed in astrocytes (Minelli et al., 1996), and because the proportion of astrocytic vs. total plasma membranes in the hippocampal neuropil is ~10% (Lehre and Danbolt, 1998), we set the density of expression of GAT3 to 10% of that of GAT1 (i.e., [*GAT3*]_*neuropil*_ = 372 μm^−3^). GATs were immobile except in the simulations described in Figure 5, where their apparent diffusion coefficient was increased up to 2 μm^2^/ms, comparable with the lateral diffusion coefficient of various neurotransmitter receptors and transporters (Heine et al., 2008; Levi et al., 2008; Bannai et al., 2009; Chamma et al., 2013).

## Changes in GABA transporter expression alter the GABA concentration profile away from the synaptic cleft

**Figure 3:**
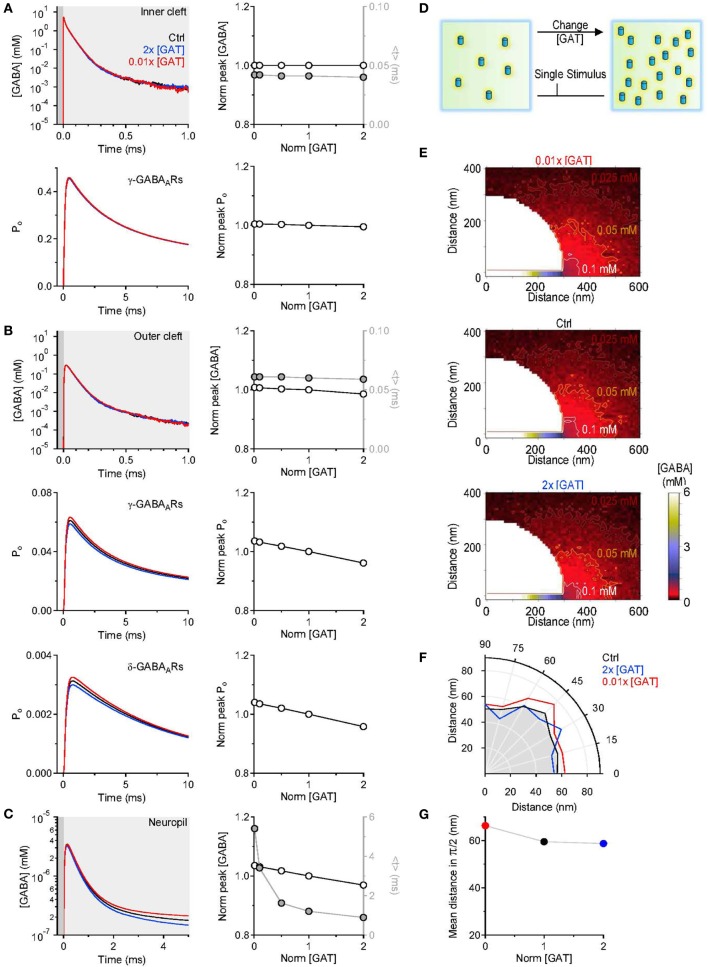
**Changing the concentration of GABA transporters alters the lifetime of GABA outside the synaptic cleft. (A)** GABA concentration profile in the inner cleft volume measured when varying the concentration of GABA transporters in the entire simulation environment (top left). The peak of the GABA concentration was normalized by its value in control conditions (*left axis and white symbols*). The normalized peak and the centroid (<*t*>, *right axis and gray symbols*) of the GABA concentration profile in the inner cleft volume are not altered when varying the density of expression of GABA transporters (top right). No change is observed in the time peak of the open probability of *γ*-subunit containing GABA_A_ receptors activated by the GABA concentration profile in the inner cleft volume (bottom right). **(B)** As in **(A)**, but the GABA concentration profile is measured in the outer cleft volume. The GABA concentration profile in the outer cleft volume is marginally influenced by changes in the density of expression of GABA transporters. This causes a progressive reduction in the activation of *γ*- and *δ*-subunit containing GABA_A_ receptors. **(C)** GABA concentration profile in the neuropil (left). Increasing the concentration of GABA transporters in the entire simulation environment leads to a small reduction in the peak and to a pronounced decrease in the lifetime of extracellular GABA. Right: the centroid of the GABA concentration profile becomes progressively smaller at higher concentration levels of GABA transporters (<*t*>, *right axis and gray symbols*); this effect is associated with a small decrease in the peak GABA concentration (*left axis and white symbols*). **(D)** Schematic diagram illustrating the change in GABA transporter concentration performed in the simulations analyzed in this figure. Each simulation involved a single release event. **(E)** Profile of the average GABA concentration measured in the neuropil surrounding the active GABAergic pre-synaptic terminal. The panels illustrate the distribution of the mean GABA concentration observed when varying the control GABA transporter concentration (middle) from 0.01 times (top) to 2 times its value in control conditions (bottom). The white, orange, and brown contours define the regions where the GABA concentration reached values of 0.1, 0.05, and 0.025 mM, respectively. **(F)** The polar graph illustrates the change in the spatial spread of the mean GABA concentration observed when varying the concentration of GABA transporters in the entire neuropil. The lines plotted in the graph were obtained by measuring the distance between the edge of the synapse and the 0.1 mM line shown in **(E)**. **(G)** Average distance between the edge of the synaptic cleft and the 0.1 mM line shown in **(E)**, for all the 0-π /2 angles analyzed in the polar plot shown in **(F)**.

In the first set of simulations, we asked how changing the density of expression of GABA transporters alters the GABA concentration profile in the volume of the inner cleft (where GABA is released; Figure 1B left), the outer cleft (the portion that surrounds the site of GABA release; Figure 1B right) and in the neuropil (the portion of the simulation environment that is not occupied by the pre-synaptic terminal and the soma; Figure 1A left). The concentration of GABA transporters (GATs) was varied between 0.01 and 2 times the value used to describe the concentration of GABA transporters in control conditions (Chiu et al., 2002; Table 1). To quantify the effects of these manipulations, we calculated the peak and the centroid of the GABA concentration profile (<*t*>). The centroid is defined as:

(2) $$<t>=\frac{\int_{0.05F{\left(t\right)}_{Max}}^{0.05F{\left(t\right)}_{Max}}t\cdot F(t)dt}{\int_{0.05F{\left(t\right)}_{Max}}^{0.05F{\left(t\right)}_{Max}}F(t)dt},$$

where *F*(*t*) represents the time course of the GABA concentration profile averaged across all simulations and *t* represents time (see also Diamond, 2005; Scimemi et al., 2009). The centroid represents the center of mass of *F*(*t*), or the average position, in time, of all the points in *F*(*t*). It is calculated over a time window that corresponds to 0.05 of the peak of *F*(*t*) [*F*(*t*)_*Max*_], before and after its onset. The results presented in Figure 3 indicate that varying the concentration of GABA transporters had no effect on the GABA concentration profile in the inner cleft volume: there was no change in the peak and in the centroid of the GABA concentration profile (Figure 3A top). As expected, this led to no change in the open probability (*P*_*o*_) of γ-subunit containing GABA_A_ receptors in the inner cleft area (Figure 3A). The effect on the GABA concentration profile in the outer cleft volume was modest, but led to a small progressive decline in the activation of γ- and δ-subunit containing GABA_A_ receptors in this region (Figure 3B). When monitoring the GABA concentration profile in the neuropil, we observed a small, progressive reduction in the peak and a significant decrease in the centroid of the GABA concentration profile at higher GABA transporter concentrations (Figure 3C). We used these simulations to derive a spatial map of GABA diffusion from its point of release toward the surrounding neuropil (Figure 3E). Consistent with the previous data, lowering the expression of GABA transporters allowed GABA to diffuse further away from the active synapse (Figures 3F,G). These findings remained qualitatively unaltered when taking into account the presence of a tonic extracellular GABA concentration of 160 nM (Santhakumar et al., 2006) (data not shown). This is probably not surprising, because this concentration is significantly lower than the substrate steady-state affinity of GABA transporters for GABA (Bicho and Grewer, 2005). The data indicate that the main effects of altering the expression of GABA transporters are detected at a distance from an active synapse. It is the activation of GABAreceptors away from the release site—not of those directly opposed to it—that can be regulated by changing the density of expression of GABA transporters. The proportion of GABA molecules that can be bound by GABA transporters in the cleft is small. This is consistent with the notion that the activation of receptors in the immediate vicinity of an active release site is dominated by diffusion, not by the activity of transporters (Rusakov and Kullmann, 1998; Barbour, 2001; Scimemi and Beato, 2009; Scimemi et al., 2009). Notably, the notion holds even at GABAergic synapses, where the neurotransmitter transporters are expressed also in the synaptic cleft region (not only at the edge of it as it happens for glutamatergic synapses He et al., 2000).

**Figure 4:**
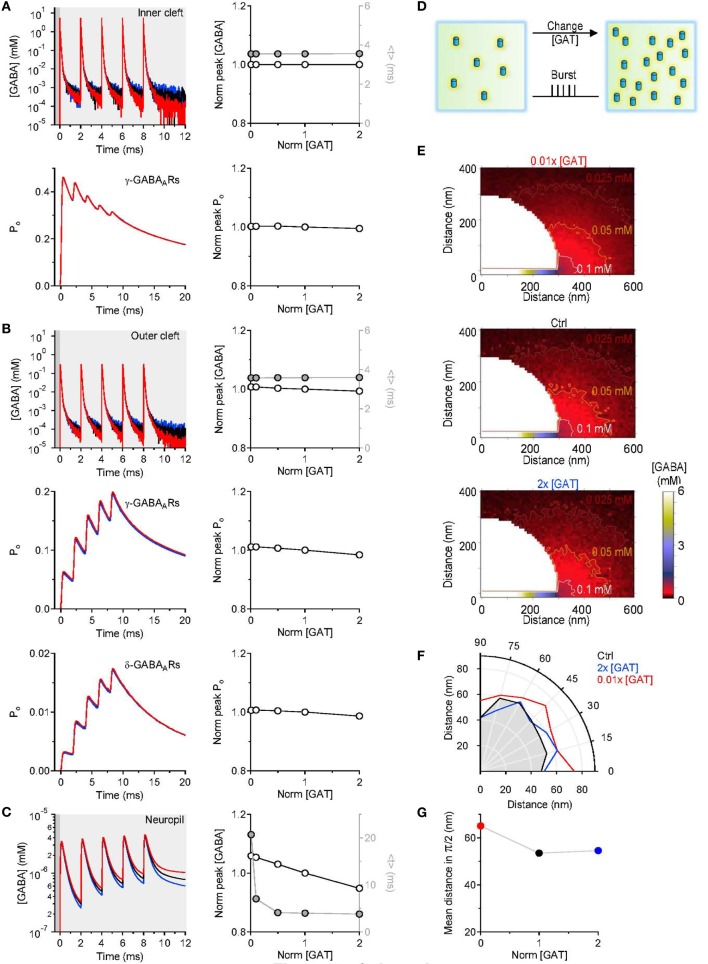
**Simulations results obtained with repeated GABA release events. (A)** Five consecutive GABA release events, 2 ms apart from each other, were simulated. The GABA concentration profile in the inner cleft volume was measured when varying the concentration of GABA transporters in the entire simulation environment (top left). The peak corresponded to the maximum GABA concentration evoked by the repeated stimuli and was normalized by the value measured in control conditions. The normalized peak (*left axis and white symbols*) and the centroid (*<t>, right axis and gray symbols*) of the GABA concentration profile in the inner cleft volume are not altered when varying the density of expression of GABA transporters (top right). The open probability (*P*_*o*_) of *γ*-subunit containing GABA_A_ receptors in the inner cleft region declines progressively with each GABA release event. The peak of the open probability of *γ*-subunit containing GABA_A_ receptors does not change when varying the concentration of GABA transporters (bottom right). **(B)** As in **A**, but the GABA concentration profile is measured in the outer cleft volume. The repeated GABA release events lead to a progressive increase in the open probability of *γ*-subunit containing GABA_A_ receptors in the outer cleft area. The GABA concentration profile in the outer cleft volume is marginally influenced by changes in the density of expression of GABA transporters. This causes minor changes in the activation of *γ*- and *δ*-subunit containing GABA_A_ receptors in the outer cleft area. **(C)** GABA concentration profile in the neuropil (left). Increasing the concentration of GABA transporters in the entire simulation environment leads to a progressive reduction in the lifetime and peak concentration of extracellular GABA. Right: the centroid of the GABA concentration profile becomes progressively smaller at higher GABA transporters concentrations (<*t*>, *right axis and gray symbols*); this effect is associated with a small decrease in the peak GABA concentration (*left axis and white symbols*). In these simulations, the centroid of the GABA concentration profile in the neuropil is calculated over a time window that corresponds to 0.20 of the peak of *F*(*t*) [*F*(*t*)_*Max*_], before and after its onset. **(D)** Schematic diagram illustrating the change in GABA transporter concentration performed in the simulations analyzed in this figure. Five release events, separated by 2 ms intervals, were simulated. **(E)** Profile of the average GABA concentration measured in the neuropil surrounding the active GABAergic pre-synaptic terminal. The panels illustrate the distribution of the mean GABA concentration observed when varying the control GABA transporter concentration (middle) from 0.01 times (top) to 2 times its value in control conditions (bottom). The white, orange and brown contours define the regions where the GABA concentration reached values of 0.1 mM, 0.05 mM and 0.025 mM, respectively. **(F)** The polar graph illustrates the change in the spatial spread of the mean GABA concentration observed when varying the concentration of GABA transporters in the entire neuropil. The lines plotted in the graph were obtained by measuring the distance between the edge of the synapse and the 0.1 mM line shown in **(E)**. **(G)** Average distance between the edge of the synaptic cleft and the 0.1 mM line shown in **(E)**, for all the 0-π /2 angles analyzed in the polar plot shown in **(F)**.

GABAergic interneurons in the hippocampus can fire bursts of action potentials and in some cases each action potential can evoke the release a synaptic vesicle (Freund and Buzsaki, 1996). We asked whether the results of our simulations would be different when multiple GABA release events are triggered from the pre-synaptic active zone. To address this, we simulated a burst of five release events, 2 ms apart from each other (Figure 4). Each time, 2000 GABA molecules were released from the center of the synapse in the synaptic cleft. Even in this case, varying the density of expression of GABA transporters did not induce any significant change in the peak and time course of the GABA concentration profile in the inner cleft volume (Figure 4A top). The peak GABA concentration in the inner cleft volume increased to ~5.3 mM with each release event. This caused progressive desensitization of γ-subunit containing GABA_A_ receptors in the inner cleft region, at all tested levels of GABA transporter expression (Figure 4A bottom). The GABA concentration profile in the outer cleft region was also not significantly affected by changes in the GABA transporter concentration (Figure 4B). Here, the activation of *γ*- and *δ*-subunit containing GABA_*A*_ receptors increased progressively with consecutive release events, and was insensitive to changes in GABA transporter concentration (Figure 4B). Similarly to what observed with single stimulations, increasing the expression of GABA transporters caused a small reduction in the peak and a profound decrease in the lifetime of extracellular GABA (Figure 4C). Consistent with these findings, the spatial maps of GABA diffusion showed that GABA diffused at higher concentration, further away from the release site when lowering the expression of GABA transporters (Figures 4E–G). A GABA transporter density of expression of 650 μm^−2^ (Chiu et al., 2002) and a pre-synaptic apposition area of 0.28 μm^2^ (Table 1) result in the presence of 182 GABA transporter molecules in the pre-synaptic membrane within the cleft region. The majority of the synaptically-released GABA molecules diffuse away from the synaptic cleft before they are bound by the transporters. Therefore, GABAergic transmission mediate by receptors located at the center of the synapse is preserved regardless of the expression levels of GABA transporters.

## Changes in GABA transporter lateral mobility alters the spatial spread, not the lifetime of GABA outside the synaptic cleft

**Figure 5:**
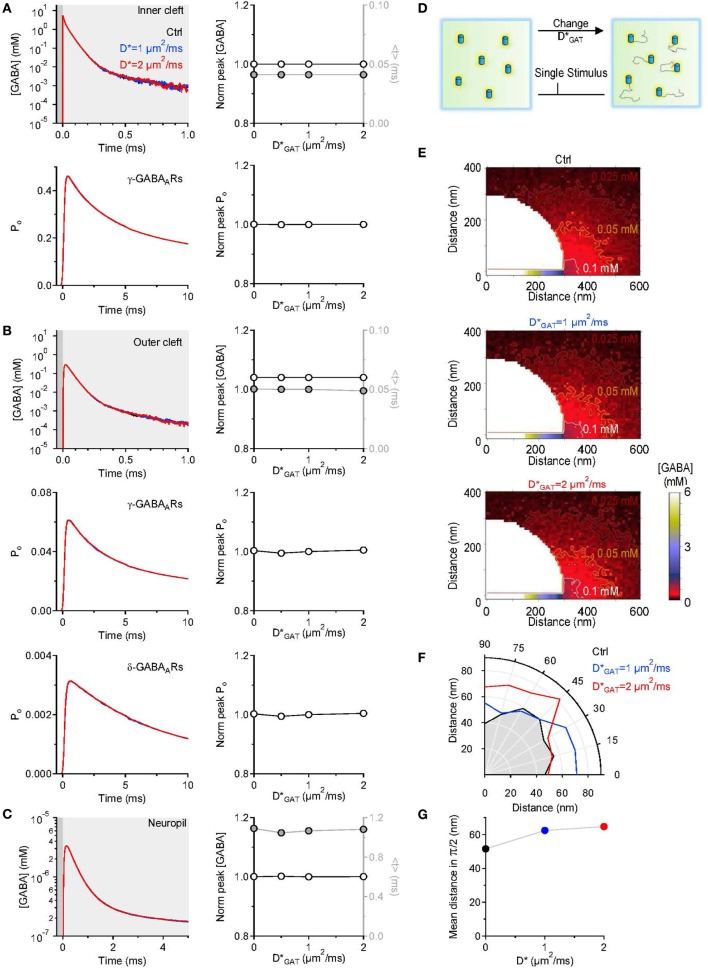
**Changing the lateral mobility of GABA transporters alters spatial spread but not the lifetime of GABA outside the synaptic cleft**. **(A)** GABA concentration profile in the inner cleft volume measured when varying the lateral mobility of GABA transporters in the entire simulation environment (top left). The centroid (<*t*>) of the GABA concentration profile in the inner cleft volume is not altered when varying the lateral mobility of GABA transporters (top right). No change is observed in the time course (bottom left) and peak (bottom right) of the open probability of *γ*-subunit containing GABA_*A*_ receptors activated by the GABA concentration profile in the inner cleft volume. **(B)** As in **(A)**, but the GABA concentration profile is measured in the outer cleft volume. The GABA concentration profile in the outer cleft volume is not influenced by changes in the lateral mobility of GABA transporters. This leads to no change in the activation of *γ*- and *δ*-subunit containing GABA_A_ receptors. **(C)** GABA concentration profile in the neuropil (left). Increasing the lateral mobility of GABA transporters in the entire simulation environment does not alter the lifetime of GABA in the extracellular space. The peak concentration (*left axis and white symbols*) and the centroid of the GABA concentration profile (*right axis and gray symbols*) are not affected by increasing the lateral mobility of GABA transporters (right). **(D)** Schematic diagram illustrating the change in GABA transporter apparent diffusion coefficient (D^*^_GAT_) performed in the simulations analyzed in this figure. **(E)** Profile of the average GABA concentration measured in the neuropil surrounding the active GABAergic pre-synaptic terminal. The panels illustrate the distribution of the mean GABA concentration observed when varying the GABA transporter lateral mobility from 0 (top) to 1 μm^2^/ms (middle) and 2 μm^2^/ms (bottom). The white, orange and brown contours define the regions where the GABA concentration reached values of 0.1, 0.05, and 0.025 mM, respectively. **(F)** The polar graph illustrates the change in the spatial spread of the mean GABA concentration observed when varying the lateral mobility of GABA transporters in the entire neuropil. The lines plotted in the graph were obtained by measuring the distance between the edge of the synapse and the 0.1 mM line shown in **(E)**. **(G)** Average distance between the edge of the synaptic cleft and the 0.1 mM line shown in **(E)**, for all the 0-π/2 angles analyzed in the polar plot shown in **(F)**.

In the simulations described above, we assumed GABA transporters to be completely immobile within the cell membrane. There is experimental evidence that indicates that there are adapter proteins, like ezrin, that anchor GABA transporters in the plasma membrane to the cell cytoskeleton (Imoukhuede et al., 2009; Moss et al., 2009). The proportion of immobile GABA transporters represents 50% of the entire population of surface GABA transporters (Imoukhuede et al., 2009; Moss et al., 2009). At steady-state, an increase in the lateral mobility of GABA transporters is associated with increased GABA uptake (Imoukhuede et al., 2009). Synaptic transmission is not a steady-state event, and the functional implications of changes in the mobility of GABA transporters are incompletely understood. To resolve this issue, in a separate set of simulations, we varied the diffusion coefficient of GABA transporters within the cell membrane and tested the effect that this had on the GABA concentration profile at different distances from an active release site. The diffusion coefficient was increased from 0 to 2 μm^2^/ms, a value that is consistent with the estimated diffusion coefficient of other neurotransmitter transporters and receptors (Heine et al., 2008; Levi et al., 2008; Bannai et al., 2009; Chamma et al., 2013). We found that increasing the lateral mobility of GABA transporters did not affect the peak and time course of the GABA concentration profile and the activation of GABA receptors in the inner (Figure 5A) and outer cleft (Figure 5B). The lifetime of GABA in the neuropil was also unaltered (Figure 5C). The only effect that we could detect was that GABA diffused further away from its release site when GABA transporters were mobile (Figures 5E–G). Therefore, increasing the mobility or the proportion of mobile surface GABA transporters facilitates GABA diffusion away from the synaptic cleft. This effect is likely to become more pronounced if the mobile GABA transporters have: (1) rapid binding and slow unbinding kinetics; (2) high lateral diffusion coefficient; (3) low transport efficiency. Under these conditions, the lifetime of the GABA-bound state would be longer than the time required for the lateral diffusion of GABA transporters away from the synaptic cleft and this could contribute to degrade the spatial specificity of GABAergic synaptic transmission.

## Conclusions

GABA transporters are expressed in neurons and astrocytes, but in most regions of the brain they reach the highest levels of expression in neuronal pre-synaptic terminals (Zhou and Danbolt, 2013). The naïve intuition is that this location is perfectly tailored to remove GABA from the synaptic cleft immediately after its release, allowing for rapid neurotransmitter recycling into pre-synaptic terminals during repeated stimulations. To date, it has not been tested whether this hypothesis holds given the rapid kinetics of neurotransmitter diffusion in the extracellular space and the binding and translocation kinetics of GABA transporters (Bicho and Grewer, 2005). Here we used a series of 3D Monte Carlo reaction-diffusion simulations to determine the effect of varying GABA transporter density of expression and surface mobility on the GABA concentration profile and the recruitment of GABA receptors at different distances from an active release site. Our findings indicate that altering surface expression and mobility of GABA transporters do not lead to changes in the GABA concentration profile in the inner portion of the synaptic cleft. In contrast, the lifetime of GABA in the neuropil surrounding an active GABAergic synapse is prolonged by reducing the density of expression of GABA transporters. Increasing the lateral mobility of GABA transporters facilitates GABA diffusion away from the synaptic cleft. These results are conceptually important because they indicate that the activation of synaptic receptors is not affected by the presence of synaptic GABA transporters. The activity-dependent regulation of intracellular signaling cascades that control the surface expression and mobility of GABA transporters mainly affect the recruitment of extrasynaptic—not synaptic—GABA receptors. Therefore, PKC and tyrosine kinase, which control the cytoplasm/cell membrane partitioning of GABA transporters, cam modify the spatial spread of GABAergic signals. These findings suggest novel mechanisms to tune the plasticity and spatial specificity of GABAergic synaptic transmission in the brain.

### Conflict of interest statement

The author declares that the research was conducted in the absence of any commercial or financial relationships that could be construed as a potential conflict of interest.
